# Strengthening health management information systems for Chagas disease: a multi-level qualitative study in Bolivia and Paraguay

**DOI:** 10.1016/j.lana.2026.101514

**Published:** 2026-05-30

**Authors:** Malia Skjefte, Elizabeth Posada, Leonardo de la Torre, Sofía Ardiles Ruesjas, Irene Losada Galván, Wilma Chambi, Miryam Rosalía Soliz Zarate, Vidalia Lesmo, Eduardo Rueda, Mayda Morales, Richard James Maude, Mirko Rojas-Cortez, Julio Alonso-Padilla

**Affiliations:** a Centre for Tropical Medicine and Global Health, Nuffield Department of Medicine, University of Oxford, Oxford, United Kingdom; b Mahidol Oxford Tropical Medicine Research Unit, Faculty of Tropical Medicine, Mahidol University, Bangkok, Thailand; c Barcelona Institute for Global Health (ISGlobal), Barcelona, Spain; d Institute of Medical Microbiology and Hygiene, Saarland University, Homburg, Germany; e Fundación Salud Naturaleza Integral - SANIT, Cochabamba, Bolivia; f Ministerio de Salud Pública y Bienestar Social-XV Región Sanitaria, Presidente Hayes, Paraguay; g Servicio Nacional de Erradicación del Paludismo (SENEPA), Programa Nacional Control de la Enfermedad de Chagas, Asunción, Paraguay; h Programa de Chagas, Servicio Departamental de Salud (SEDES)- Tarija, Bolivia; i Programa de Chagas, Servicio Departamental de Salud (SEDES)- Santa Cruz, Bolivia; j CIBER de Enfermedades Infecciosas, Instituto de Salud Carlos III (CIBERINFEC, ISCIII), Madrid, Spain; k Facultat de Medicina i Ciències de la Salut, Universitat de Barcelona (UB), Barcelona, Spain; l The Open University, Milton Keynes, United Kingdom

**Keywords:** Chagas disease, *Trypanosoma cruzi*, Routine surveillance data, Health management information system, Bolivia, Paraguay, The Chaco

## Abstract

**Background:**

Chagas disease is a neglected tropical disease caused by the parasite *Trypanosoma cruzi*. It causes a significant health burden in the Americas, especially in Bolivia and Paraguay, where the disease is most endemic in the transnational Chaco region. Issues with data quality and availability prevent a true understanding of the current burden of disease, with missed opportunities for surveillance, resource allocation, and evaluation of interventions. This study aimed to explore routine Chagas disease data systems in Bolivia and Paraguay, focusing on data flow, barriers to data quality and reporting, and data sharing across health system levels, and to identify opportunities to strengthen surveillance and decision-making.

**Methods:**

A qualitative study was conducted between July–September 2024 in Bolivia and Paraguay. In-depth interviews were carried out with 43 key informants, with roles as healthcare workers, data managers, and technical advisors involved in Chagas Programs across different health system levels. A thematic analysis was conducted to develop a set of themes and recommendations related to data management practices.

**Findings:**

Four key themes emerged throughout interviews, with findings on data management practices, barriers to high quality routine data, data use, and cross-border data sharing. Key recommendations from participants to improve the overall Chagas disease data management systems in each country included strengthening digital infrastructure, promoting interoperability across systems, and fostering regional collaboration for cross-border data exchange.

**Interpretation:**

The study highlights critical gaps in routine data management for Chagas disease and underscores the importance of national-level solutions to support surveillance and control efforts. These findings offer concrete, actionable guidance for national health authorities and international stakeholders working to improve data systems for this neglected disease in endemic regions.

**Funding:**

This work was supported by the 10.13039/100022401Fulbright U.S. Student Program; the 10.13039/100010269Wellcome Trust [220211/Z/20/Z]; and 10.13039/501100024035ISGlobal (CEX2023-0001290-S, MCIN/AEI/10.13039/501100011033), with additional support from the 10.13039/501100002809Generalitat de Catalunya (CERCA Program).

## Abstract (es)

**Introducción:**

La enfermedad de Chagas es una enfermedad tropical desatendida causada por el parásito *Trypanosoma cruzi*. Representa una importante carga sanitaria en las Américas, especialmente en Bolivia y Paraguay, donde la enfermedad es más endémica en la región transnacional del Chaco. Los problemas relacionados con la calidad y disponibilidad de los datos impiden una comprensión real de la carga actual de la enfermedad, lo que limita las oportunidades para la vigilancia, la asignación de recursos y la evaluación de intervenciones.

**Métodos:**

Se realizó un estudio cualitativo entre julio y septiembre de 2024 para comprender el flujo actual de los datos rutinarios de la enfermedad de Chagas desde el nivel local hasta el nivel nacional en Bolivia y Paraguay, e identificar las principales barreras para una gestión de datos de calidad en cada país. En total, se entrevistó a 43 participantes en ambos países, incluyendo personal sanitario, gestores de datos y asesores técnicos de los programas de Chagas. Los datos de las entrevistas se analizaron mediante un análisis temático para desarrollar un conjunto de temas y recomendaciones.

**Resultados:**

Se identificaron cuatro temas clave a partir de las entrevistas, relacionados con las prácticas de gestión de datos, las barreras para obtener datos rutinarios de alta calidad, el uso de los datos y el intercambio transfronterizo de información. Las principales recomendaciones de los participantes para mejorar los sistemas de gestión de datos de la enfermedad de Chagas incluyeron el fortalecimiento de la infraestructura digital, la promoción de la interoperabilidad entre sistemas y el fomento de la colaboración regional para el intercambio de datos entre países.

**Interpretación:**

El estudio pone de manifiesto brechas críticas en la gestión de datos rutinarios sobre la enfermedad de Chagas y destaca la importancia de soluciones a nivel nacional para apoyar los esfuerzos de vigilancia y control. Estos hallazgos ofrecen orientaciones concretas y aplicables para las autoridades sanitarias nacionales y los actores internacionales que trabajan para mejorar los sistemas de datos de esta enfermedad desatendida en regiones endémicas.

**Financiación:**

Este trabajo fue financiado por el programa Fulbright U.S. Student; el Wellcome Trust [220211/Z/20/Z]; e ISGlobal (CEX2023-0001290-S, MCIN/AEI/10.13039/501100011033), con apoyo adicional de la Generalitat de Catalunya (Programa CERCA). Con el propósito de acceso abierto, el autor ha aplicado una licencia pública CC BY a cualquier versión del manuscrito aceptado derivada de esta presentación.

## Resumo

**Introdução:**

A doença de Chagas é uma doença tropical negligenciada causada pelo parasita *Trypanosoma cruzi*. Representa uma importante carga de doença nas Américas, especialmente na Bolívia e no Paraguai, onde a doença é mais endêmica na região transnacional do Chaco. Problemas relacionados à qualidade e disponibilidade dos dados dificultam uma estimativa precisa da carga atual da doença, limitando oportunidades para vigilância, alocação de recursos e avaliação de intervenções.

**Métodos:**

Foi realizado um estudo qualitativo entre julho e setembro de 2024 para compreender o fluxo atual dos dados rotineiros da doença de Chagas desde o nível local até o nível nacional na Bolívia e no Paraguai, e identificar as principais barreiras para uma gestão de dados de qualidade em cada país. Foram entrevistados, 43 participantes nos dois países, incluindo profissionais de saúde, gestores de dados e assessores técnicos dos programas de Chagas. Os dados das entrevistas foram analisados por meio de análise temática para desenvolver um conjunto de temas e recomendações.

**Resultados:**

Quatro temas principais emergiram das entrevistas, relacionados às práticas de gestão de dados, barreiras à obtenção de dados rotineiros de alta qualidade, uso dos dados e compartilhamento transfronteiriço de informações. As principais recomendações dos participantes para melhorar os sistemas de gestão de dados da doença de Chagas incluíram o fortalecimento da infraestrutura digital, a promoção da interoperabilidade entre sistemas e o incentivo à colaboração regional para o intercâmbio de dados entre países.

**Interpretação:**

O estudo destaca lacunas críticas na gestão de dados rotineiros sobre a doença de Chagas e ressalta a importância de soluções em nível nacional para apoiar esforços de vigilância e controle. Esses achados oferecem orientações concretas e aplicáveis para autoridades de saúde nacionais e atores internacionais que trabalham para melhorar os sistemas de dados dessa doença negligenciada em regiões endêmicas.

**Financiamento:**

Este trabalho foi financiado pelo programa Fulbright U.S. Student; pelo Wellcome Trust [220211/Z/20/Z]; e pelo ISGlobal (CEX2023-0001290-S, MCIN/AEI/10.13039/501100011033), com apoio adicional da Generalitat de Catalunya (Programa CERCA). Para fins de acesso aberto, o autor aplicou uma licença pública CC BY a qualquer versão do manuscrito aceito resultante desta submissão.

**Research in context**

**Evidence before this study**

Before conducting this study, we searched PubMed and Google Scholar for relevant articles published between January 1, 2000 and June 30, 2024 using combinations of the following terms: “Chagas disease” OR “Trypanosoma cruzi” AND “surveillance” OR “routine data” OR “health information system” OR “health management information system” OR “HMIS” OR “data quality” OR “data management” OR “Bolivia” OR “Paraguay” OR “Gran Chaco”. We also reviewed relevant technical reports and articles from multilateral organizations, including the World Health Organization (WHO) and other international groups, for any documents on routine surveillance systems for neglected tropical diseases with a focus on Chagas. We did not restrict the search by language.

We found that existing literature on Chagas disease highlights persistent underdiagnosis of the disease, fragmented surveillance structures, and limited integration of entomological, clinical, and laboratory data. Additionally, we found that most available evidence focuses on topics such as prevalence estimates, diagnostic performance, treatment outcomes, or modeling of disease burden rather than on the routine flow of data across the health system. While we did not find any qualitative studies examining routine Chagas disease data management and surveillance in endemic countries, some papers presented the need for cross-border data exchange for this disease. Overall, the available evidence suggests structural weaknesses in Chagas surveillance but provides limited understanding of how frontline actors experience and navigate these systems in endemic settings.

**Added value of this study**

Our study provides a comprehensive, multi-level qualitative assessment of routine Chagas disease data management in two highly endemic countries in the Americas: Bolivia and Paraguay. Through interviewing 43 participants across each level of the healthcare system (from the primary health facility to interviews with national authorities), we map the full routine data lifecycle and center our findings grounded in the lived experiences of those managing Chagas data on a day-to day basis. Additionally, this study highlights shared regional challenges, drawing attention to the need for standardized indicators and interoperable data platforms to support cross-country collaboration in endemic countries. To the best of our knowledge, this is one of the first studies to examine routine Chagas surveillance systems through a comparative, cross-border lens in Bolivia and Paraguay.

**Implications of all the available evidence**

Taken together with existing literature on Chagas burden, surveillance gaps, and neglected tropical disease prioritization, our findings underscore the urgent need for investment in integrated, digital, and interoperable health management information systems for Chagas disease in endemic countries. Essential to achieving the 2030 targets for the elimination of Chagas disease as a public health problem in the Americas, policymakers and international stakeholders should prioritize the development of a centralized HMIS platform for this neglected disease. Additional priorities include the need for standardized core indicators, improved laboratory and blood bank integration, enhanced training and resourcing at the local level, and stronger feedback mechanisms across health system levels. At the regional level, stakeholders can also work towards advancing structured cross-border data sharing frameworks. Future research can explore the topic of Chagas disease data management in other bordering endemic countries, as well as evaluate the feasibility of digital and policy reforms aimed at improving routine Chagas surveillance.

## Introduction

Chagas disease (CD) is a neglected tropical disease (NTD) caused by the protozoan parasite *Trypanosoma cruzi* (*T. cruzi*) with an estimated seven million people infected and 100 million at risk in 2018.^1^^,^^2^ Although global CD prevalence has decreased by 16.1% over the last three decades (1990–2019), the disease was responsible for approximately 8400 deaths and 352,000 new cases globally in 2023, according to Global Burden of Disease (GBD).^3^ The true burden of CD may be grossly underestimated, with estimation challenges due to the delayed presentation of symptoms, serosurvey limitations, variability in transmission, and challenges with surveillance systems and access to diagnosis, among other factors.^2^ According to the Pan American Health Organization (PAHO), for example, it is estimated that less than 10% of people infected with *T. cruzi* have been diagnosed, and even fewer have received treatment for the disease.^4^ Therefore, there is an urgent need for comprehensive and high quality routine data, collected over a sustained period of time, to more accurately quantify the true burden of *T. cruzi* infections.

Latin America suffers from the highest burden of CD, due to the presence of the primary vectors (infected triatomine bugs) and socioeconomic factors that contribute to disease transmission. With approximately 6·9% of the population infected with *T. cruzi*, Bolivia has the highest estimated prevalence rate.^1^^,^^5^ To date, several studies have reported a high prevalence of CD in the Chaco region, a semi-arid lowland spanning the border between Bolivia and Paraguay (also including a vast area of Northern Argentina), that poses difficulties to vector control and surveillance.^6^, ^7^, ^8^ Paraguay also has a high estimated prevalence of CD, especially among indigenous populations which are disproportionately affected by the disease. While the country has been successful in interrupting transmission of *T. cruzi* infection by one species of the vector (*Triatoma infestans*), estimates show that approximately 150,000 people are *T. cruzi-*infected with continued threats from other vector species responsible for transmission.^9^ In a 2023 seroprevalence study in Casanillo, located in the Paraguayan Chaco (Presidente Hayes department), the seroprevalence for *T. cruzi* infection was 12·6% amongst a sample of 999 participants, compared to a national prevalence of 2·1%.^10^ These findings highlight the need for additional routine data to be collected to have a more accurate understanding of the true burden of the disease.

Targeted seroprevalence studies have helped researchers and decision-makers understand CD prevalence at specific points in time; however, there remains a need for high quality, consistent, routine surveillance data to identify trends and inform public health responses. According to the World Health Organization (WHO), only six out of 44 countries that report CD cases have a national health management information system (HMIS) to monitor acute and chronic cases as well as active transmission routes.^11^ Many studies have stressed the importance of a strong, centralized HMIS, including its use in delivering health services and generating high quality data for disease program decision making.^12^^,^^13^ Without integrated routine CD data systems, countries face significant challenges in evaluating intervention effectiveness and allocating resources for disease control and elimination efforts. While centralized HMIS platforms, such as District Health Information System 2 (DHIS2) and National Malaria Data Repository (NMDR) platforms, both widely used across low- and middle-income countries (LMICs), have improved real-time data access and decision-making in infectious disease programs like those for malaria,^14^ the absence of comparable integration for CD contributes to fragmented systems and delayed responses. Furthermore, as disease transmission dynamics are shaped by factors that transcend national boundaries and complicate coordinated surveillance and response efforts, such as vector ecology, climate change, and human mobility,^15^^,^^16^ addressing these gaps through more integrated data systems could therefore strengthen regional disease control efforts across border systems and support progress toward achieving the global target of eliminating CD as a public health problem by 2030, as outlined in the WHO NTD Roadmap.^17^

With limited literature on the topic of CD data management practices and a growing need to prioritize CD programs, this study sought to provide a set of findings and recommendations to improve the quality and availability of routine CD data in two endemic countries: Bolivia and Paraguay. Through key informant interviews (KIIs) with technical professionals working on CD across each level of the healthcare system, the study aimed to understand the flow of CD data from the local and national level, identify key barriers to quality data collection and reporting, and understand how data is shared both within and between endemic countries. Overall, this study aims to provide actionable recommendations and insights for national health authorities in Bolivia and Paraguay, while also emphasizing to infectious diseases experts the importance of high-quality routine CD data. In doing so, it addresses a critical gap in understanding how CD data are managed within routine systems and how these systems can be strengthened to better support disease surveillance and programmatic decision-making in the future.

## Methods

### Study design and setting

**Fig. 1:**
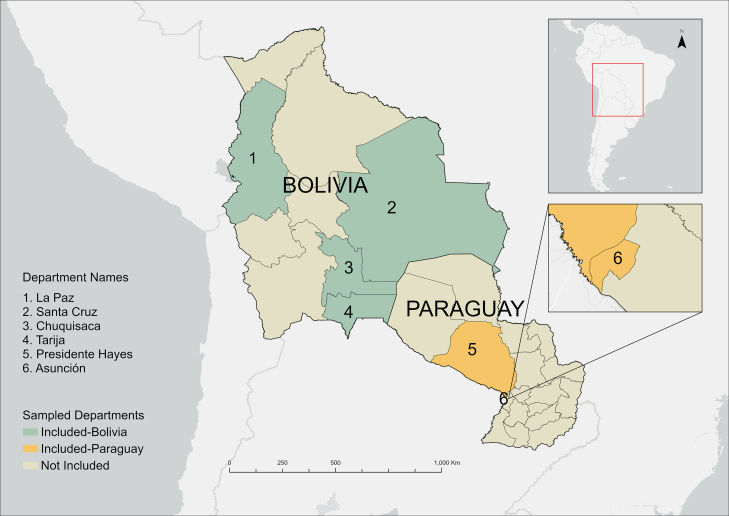
**Map of sampling locations where participants were recruited.** Subnational administrative boundary shapefiles for both countries were retrieved from the Humanitarian Data Exchange (HDX), United Nations Office for the Coordination of Humanitarian Affairs (OCHA), licensed under CC BY 4.0.

A descriptive, phenomenological qualitative study was conducted among key participants in two Chagas endemic countries: the Plurinational State of Bolivia and the Republic of Paraguay. This study design was chosen to understand how various key informants experience and perceive the data management process in CD surveillance, to uncover any barriers to quality data management in their work, and to highlight the complexities of data management systems for CD. No formal theoretical framework was applied, as the aim was to avoid constraining data collection and analysis within predefined constructs. Participants included healthcare workers (HCWs), statisticians/data managers, and technical advisors for disease programs focused solely on or providing support for CD alongside other vector-borne diseases. Interviews in Paraguay took place between July–September 2024 and in Bolivia between August–September 2024. Methods and findings have been presented according to the Tong et al. COREQ Checklist.^18^ Fig. 1 presents the sampling locations in each country.

### Participant recruitment

Participants were recruited using a purposive sampling strategy, defined as the intentional selection of information-rich participants with relevant experience related to the phenomenon explored in the study.^19^^,^^20^ This sampling strategy was selected to identify participants who had direct knowledge and experience with CD data management across different levels of the health system in the sampled countries. Through the use of a gatekeeper, the research team was connected with potential study participants in the community based on the set inclusion/exclusion criteria (Supplementary Table S1). Participants were eligible for an interview if they were ≥18 years of age, had current or recent experience working with CD data in relevant health system roles in Bolivia or Paraguay, and provided informed consent. No exclusion criteria were applied. Recruitment happened both in-person and online, most commonly through WhatsApp, a messaging platform. For each potential participant, the gatekeeper provided a brief description and purpose of the study as well as a list of the interview questions to the potential participant before they decided to participate. As the interviewer and participants did not have an established relationship prior to study commencement, a full description of the study purpose, interviewer’s role in the research team, and rationale for conducting the study were provided in the informed consent form before each interview and reviewed again during the in-person informed consent process.

The aim of the sampling was to have representation at each level of the health system, with participants from the local/facility level (clinics and hospitals), the municipality level (municipality health networks and data management touch points), the geographic departmental level (Chagas Programs and data management touch points), and the national level (Ministry of Health (MOH) and other national level groups). Participants working at reference laboratories and blood banks were also recruited for the study across levels. An additional focus was to have representation from both urban and rural locations, including participant sampling in the Chaco region.

### Data collection

To prepare the study instrument, a draft interview guide was shared with various researchers working on CD for feedback and recommendations on study questions. The interview guide was also adjusted after an initial set of three pilot interviews, which were included in the final analysis. Interviews included questions on participants’ previous experience working with CD; data collection, analysis and reporting processes; information on data sharing (within their country and with bordering countries); and lessons learned from other health areas. A list of interview topics is provided in Table 1 with the full interview guide included in Supplementary Table S2, which was used as a standard guide across interviews. Additional probing questions were provided to participants based on health system level and role.

**Table 1:** Interview guide topics for study participants.

Participant background
• Profession and training • Description of current role and day-to-day tasks • Number of years working in role • Number of years working with CD data
Experiences with CD data
• Description of experiences with data collection, data management, analysis, and data sharing • Difficulties with data collection, data management, analysis, and data sharing • Description of data being collected (type of data, who it is being sent to, and with what frequency)
Data collection and reporting forms
• Familiarity with forms to collect data and notify cases • Overview of strengths and limitations of form(s) • Recommendations to improve form
Integration with other health areas
• Description of experiences with other health areas/disease programs • Overview of data flow for other health areas/disease programs • Lessons that can be drawn from other health areas/disease programs to improve data management for CD • Ways to better integrate CD data management into an overall reporting system in the country
Data exchange (within and between countries)
• Description of data flow within country • Description of data sharing with other endemic, bordering countries • Steps and resources necessary to create a strong data sharing system between countries • Type of information most valuable to share between countries (i.e., clinical data, entomological data, stakeholder learnings)
Conclusion/final thoughts
• Final thoughts or recommendations on how to strengthen CD data management in the country

All interviews were audio-recorded, with in-person interviews recorded on a mobile phone and virtual interviews recorded through Zoom (Version 6.0). Each interview lasted on average 34·6 min (range 14–64 min), and they were conducted by one female researcher (MS) who took notes throughout the interview. For in-person interviews, participants selected the location for the interview, usually their work office or personal home, to create the most privacy. While emphasis was placed on ensuring a private and quiet environment, some interviews took place in front of coworkers or family members. No repeat interviews were carried out during the study.

### Data saturation

Participant recruitment was finalized once there was representation from each health system level for each country. Code saturation was reached during the interviews in both countries, as there were no additional themes or insights that emerged from the data, and all relevant conceptual categories were identified, explored, and exhausted.

### Data analysis

To prepare for the data analysis, all recordings were transcribed and translated into English using online software (Otter Ai and DeepL Translate, respectively). To ensure data quality, all recordings were compared with the Spanish transcription to make any additional edits before translation. An inductive-deductive approach was used for the data analysis, in which the interview guide was used to create an initial code book, with additional codes added during the coding process. Once all transcripts were confirmed and the preliminary code book was created, the study materials were uploaded into the mixed-methods analysis software Dedoose (Version 9.0) for coding and analysis. The coded excerpts were then downloaded by major code, and narrative summaries were formed to identify emerging themes and recommendations from the data. While transcripts were not returned for comments or corrections during the analysis process, recommendations were shared with study participants to triangulate the findings and allow for any additional insights before the analysis was finalized. Three team members conducted the coding and analysis: one researcher who conducted interviews in both countries and analyzed all transcripts (MS), and two researchers with prior experience working on CD in the sampled countries who did not participate in the interviews (EP and LT). While the coding was conducted in English, the original recordings and transcriptions in Spanish were used to confirm study results during the analysis and identify key quotes.

### Data storage and confidentiality

All data were stored in a restricted-access Google Drive folder with encryption enabled and password-protected access limited to authorized study personnel for the duration of the study. Once the interview took place, each participant was assigned a unique code used to label the transcripts and audio-recordings. Patient names were removed to de-identify the data. For each interview recorded on a mobile phone, the recording was moved to the secure Google Drive after the interview and promptly deleted from the mobile device. The recordings were then deleted once the transcripts were completed and verified to further protect the privacy of the study participants.

### Reflexivity statement

Author MS conducted the interviews and led the analysis as part of a Fulbright research project, working alongside researchers at ISGlobal and local partners SENEPA (Paraguay) and Fundación SANIT (Bolivia). She holds a Master of Science (MS) in Global Health and worked as a Fulbright Scholar at the time of the interviews. As a visitor to the study communities, MS worked with local gatekeepers in each country to support participant recruitment. Several co-authors are members of the study communities, contributing contextual knowledge and long-standing engagement with local health systems. Other authors are based at a Spanish research institute (ISGlobal) and have extensive experience working closely with partners and communities in both countries through sustained research collaborations. The research team has experience conducting qualitative research and have completed relevant training in research ethics, including ethical requirements for community-based and qualitative studies.

### Ethical considerations

Local ethical approval was received through host research institutions in both countries to conduct this work, which included SENEPA in Paraguay (CEI-LCSP N 253–2025) and the Colegio Médico de Santa Cruz in Bolivia (TEDM CITE N° 025/2024). Each participant provided written informed consent for participating in the interview. For in-person interviews, participants signed a paper consent form while participants participating through Zoom signed and shared a copy of the informed consent online.

### Role of the funding source

The funders had no role in study design, data collection, analysis, interpretation, manuscript preparation, or publication decisions.

## Results

### Participant demographics

A total of 43 participants completed interviews, including 25 from Bolivia and 18 from Paraguay (Table 2). Across countries, 16 participants were sampled at the local/facility level, six at the municipality/district level, 15 at the departmental/regional levels, and six at the national level. Participants spent, on average, 8·6 years (SD = 9·0) working in their current role with 11·9 years (SD = 9·2) working with CD data at the time of their interview. Representation was slightly higher in urban contexts, as 25 participants worked in an urban zone and 18 worked in a rural zone. Similar participant roles were sampled across health system levels in both countries; however, Bolivia had greater representation of vector control and data management roles, while Paraguay had stronger representation of frontline healthcare providers and national policy leaders. Most interviews were conducted in person (n = 37), with a smaller number conducted online (n = 6). There were an additional three participants at the national level; (one from Paraguay and two from Bolivia) who were invited to participate and refused an interview as they needed and were unable to receive permission from their employer to participate. Full participant demographics for each country are presented in Supplementary Table S3 (Bolivia) and Supplementary Table S4 (Paraguay).

**Table 2:** Summarized participant demographics for key informants across countries.

Country	Health system level	# of participants (n)	Primary organization types represented	Predominant roles	Location
Bolivia	Local/facility	8	Hospitals and clinics (secondary and tertiary)	Medical doctors, biochemists, statisticians	Yacuíba (Rural) Tarija (Urban)
	Municipality/district	5	Government offices Health networks (Red de Salud)	Public health directors, epidemiologists, statisticians, data managers	Yacuíba (Rural) Santa Cruz (Urban)
	Departmental	11	SEDES^a^, SUIS^b^, blood banks, departmental Chagas programs	Technical advisors, program directors, medical doctors, statisticians, biochemists	Tarija (Urban) Santa Cruz (Urban)
	National	1	MOH/SUIS	Medical doctor/technical advisor	La Paz (Urban)
**Subtotal**		**25**			
Paraguay	Local/facility	8	Hospitals and clinics (primary, secondary, tertiary)	Medical doctors, nurses	Asunción (Urban) Irala Fernández (Rural) Campo Aceval (Rural) El Estribo (Rural) Casanillo (Rural)
	Municipality/district	1	Hospital/clinic	Nurse/municipal data manager	Irala Fernández (Rural)
	Departmental	4	Blood banks, regional health authority, central laboratory	Medical doctors, epidemiologists, biochemists, data managers	Asunción (Urban) Presidente Hayes (Urban) Irala Fernández (Rural)
	National	5	MOH, SENEPA, central reference laboratory	Technical advisors, medical doctors, statisticians, biochemists	Asunción (Urban)
**Subtotal**		**18**			

a SEDES: Servicio Departamental de Salud or “Departmental Health Services”.

b SUIS: Sistema Único de Información en Salud or “Unified Health Information System”.

### Presentation of themes

#### Theme one: fragmented Chagas data systems across the data lifecycle

##### Data collection across data sources and levels

Across both countries, CD data collection involved multiple epidemiological, clinical, and entomological sources operating largely in parallel. At the facility level in Bolivia, clinical and epidemiological data included patient history, diagnostic testing, treatment initiation, follow-up outcomes, demographic characteristics, pregnancy status, and weekly surveillance reports of suspected and confirmed cases. While treatment status data were collected, post-visit follow-up was inconsistently tracked and varied across facilities. Entomological data related to housing infestation and vector control activities were collected separately and rarely integrated with clinical data, with most facilities relying on Excel spreadsheets or paper-based tools.

In Paraguay, facilities collected data using notification forms for both positive and negative diagnostic results, treatment status, and congenital case tracking. As in Bolivia, entomological data were typically managed separately and were not consistently used in clinical or programmatic decision-making.

Across both countries, national-level indicators were used primarily for reporting to international organizations and performance monitoring, although participants described limited training and clarity regarding indicator generation and interpretation.

##### Data flow pathways from local to national levels

In Bolivia, CD data followed a multi-step upward reporting pathway beginning at the facility level, where data were recorded using standardized Sistema Único de Información en Salud (SUIS) paper forms or local Excel logs. Positive cases were immediately communicated to higher levels via phone or WhatsApp, followed by formal paper submission. Data were aggregated at municipal or network levels and subsequently forwarded to both SUIS and departmental Chagas Programs through separate channels. SUIS data focused on notifiable diseases and aggregated surveillance indicators, while departmental Chagas Programs data emphasized individual-level clinical management and operational tracking. These parallel reporting streams were transmitted independently to national epidemiology and Chagas Program teams, with no automated linkage between systems.

**Fig. 2:**
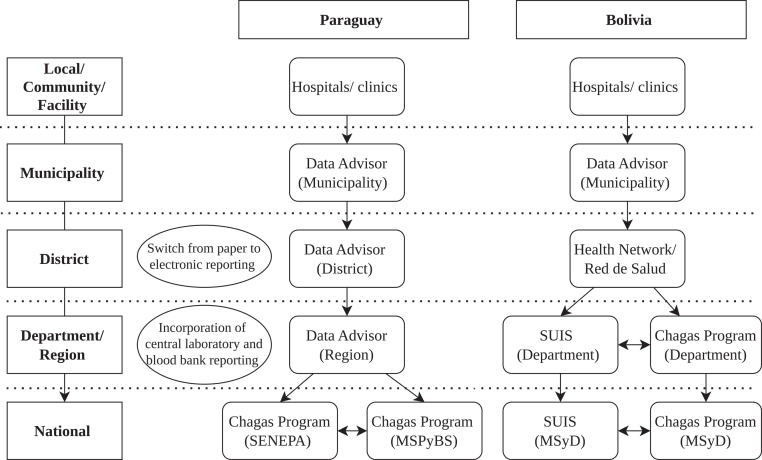
**Chagas disease surveillance data flow chart for each country (local to national level)**.

In Paraguay, facility-level data were similarly collected on paper and later digitized. Positive cases were reported immediately, while routine data were submitted monthly to regional surveillance offices. Regional teams entered data into the HMIS, called “SABER”, where information was cleaned and consolidated before submission to either the Ministerio de Salud Publica y Bienestar Social (MSPyBS) or Servicio Nacional de Erradicación del Paludismo (SENEPA). Vector control data were managed by SENEPA, while clinical and laboratory data were overseen by MSPyBS, creating partial integration at the national level but continued fragmentation across data domains. Fig. 2 presents the flow of routine CD data in each country.

##### Role of blood banks and laboratories in data collection and reporting

Blood banks and laboratories represented additional, largely siloed data sources in both countries. Blood donor screening data were centrally managed but not routinely shared with Chagas Programs, limiting opportunities for identifying and following up chronic cases. Laboratory data were often paper-based, inconsistently centralized, and shared through informal channels, contributing to delays, missing results, and limited clinical follow-up.

##### Consequences of fragmented and parallel reporting systems

Participants consistently described fragmented data systems as a major obstacle to accurate surveillance, patient follow-up, and national reporting. Parallel systems resulted in duplicated case counts, incomplete clinical histories, and difficulties producing reliable national dashboards. Weak integration between laboratories, blood banks, and clinical services further undermined comprehensive case tracking and continuity of care across both countries. Key quotes are presented in Table 3, with additional supporting quotes presented in Supplementary Table S5.

**Table 3:** Key participant quotes by theme.

Theme one: fragmented chagas data systems across the data lifecycle	*“We have protocols for the National Program and obviously for the departmental Chagas Program … but we do not have a systematized database through any program. So, it is managed with the information that [the municipality] sends … monthly information in forms.” (Departmental Level, Bolivia)*
	*“The flow of information is not systematized. We have the data and generally the information we send is according to separate requests. For example, the Chagas Program or any other Program that asks us for prevalence or the frequency of reactive units, we can send it, but there is no dynamic flow of the total epidemiological information of these units. We are working on it, but currently we do not have it.” (Regional level, Paraguay)*
Theme two: barriers to high quality routine data	*“We handle a lot of information … Since there are very few of us here, we analyze the information and we emphasize where there are more cases. All of [the disease programs] are necessary, yes, but we give more emphasis where there are more cases.” (Departmental level, Bolivia)*
	*“The files are not well filled out, we do not have complete data. Although it is true that we are improving, there is still a long way to go, but at least we are on the way. As I said, we do not have such a robust base that has all the data with quality … (National level, Paraguay)*
Theme three: data quality control, use, and feedback loops	*“The first levels detect the cases and they report it here to the network. From the network, we report it to the SEDES [Department] and from the SEDES it goes to the Ministry. That would be the way. The difficulty is that sometimes a facility handles the data manually, let's say, or by program, and does not check it against the SUIS, which is the National Information System that integrates all the information from a facility. So, there tends to be no correlation of data.” (Municipality Level, Bolivia)*
	*“Data is shared through the General Directorate of Health Surveillance. SENEPA has to report to the General Directorate of Health Surveillance and the notifications of positive cases and the interventions made in this regard. And the General Directorate of Health Surveillance is the one that actually puts out the newsletters.” (National Level, Paraguay)*
Theme four: cross-border data sharing	*“We should have one or two meetings about [a shared platform] and be able to see the usefulness, and decide what we want to see, monitor and what we want to know as well. Because the data and information is so broad, that sometimes distracts the effort … From there we can improve, expand according to the need.” (Departmental Level, Bolivia)*
	*“Perhaps a regional database or a platform where one can share this data and people who are treating these patients can have access to it … So if there was perhaps a base- a database, or a platform, or a form of communication- that would be great.” (Facility Level, Paraguay)*
Cross-cutting synthesis of participant-suggested recommendations for improving data management	*“What would be ideal would be to have at least a single set of indicators at the regional level to be able to compare data. If each country does not have their own indicators, then how can we compare ourselves? That is why it would really be ideal to have comparable data between countries, like specific indicators between countries, so that they can be exchanged and compared. And even at the country level, there is a need to define which are the most important indicators and to watch over those indicators to draw some useful conclusions.” (National level, Paraguay)*
	*“It would be interesting if countries such as the United States contributed to Latin American countries in developing technologies to better process and manage information on this disease. With adequate information, we can better see the reality we live with and make interventions—developing medicines and vaccines. Chagas has long been a forgotten disease, mainly affecting poor people in rural areas, and since it didn’t affect Europe, the U.S., or other regions, it generated little concern. Now that Chagas appears in Europe, Asia, Africa, and the U.S., more attention is being paid. These countries could help by improving computer systems and educating people to manage and process information better. Great studies could be carried out, especially on Chagas disease in Bolivia.” (Facility level, Bolivia)*

#### Theme two: barriers to high quality routine data

##### Human resource and training constraints

A lack of dedicated personnel for CD data management was a pervasive challenge, particularly at local and municipal levels. Health workers were often responsible for multiple disease programs while simultaneously providing clinical care, leading to incomplete forms, data entry errors, and delayed reporting. Participants reported limited training in data reporting procedures, use of digital tools, and statistics for required calculations, contributing to misclassification and inconsistent data quality. Participants noted previous training exchanges between countries, such as local level healthcare professionals from Paraguay visiting a municipality in Bolivia to receive training and learnings from clinic staff on how to best manage CD cases. This type of cross-border training was deemed extremely beneficial, especially for local level healthcare providers, to learn best practices and make local adaptations upon return to their communities.

##### Infrastructure, geography, and access constraints

Resource constraints such as unreliable internet connectivity, equipment shortages, and limited access to computers or paper-based forms hindered timely data entry and submission. Geographic isolation, particularly in rural and Chaco regions, was also reported by participants as a reason for delayed diagnostic confirmation and data transmission.

Cultural and linguistic differences limited data completeness. In Paraguay, for instance, health workers shared that patients from indigenous communities were sometimes reluctant to provide clinical information or blood samples, reflecting mistrust and misconceptions about the use of biological materials. These barriers contributed to underdiagnosis and underreporting, making it difficult to capture the true disease burden in these affected areas.

##### Implications for data completeness and timeliness

Incomplete follow-up, missing information, and reporting delays compromised data reliability at all system levels. At the facility level, inconsistent patient tracking was a common concern, particularly for mobile or migratory populations whose follow-up appointments were often missed. In Bolivia, the absence of nominal registries made it especially difficult to monitor patients over time, while in both countries, the deletion or loss of project data after temporary initiatives ended further hindered longitudinal tracking of treatment outcomes. Given the chronic nature of CD, these weaknesses in follow-up were seen as major obstacles to accurate surveillance and program planning.

Delays in data submission were also pervasive. Slow turnaround from under-resourced facilities often resulted in incomplete national datasets, while chronic lateness in monthly reporting cycles sometimes forced regional staff to submit partial data. Participants noted that these delays were compounded by fatigue and competing priorities among overstretched local staff. Incomplete or partially filled reporting forms were common, and information not entered into the central system at the facility level was effectively lost to national reporting. Collectively, these delays and data gaps disrupted the flow of information needed for timely decision-making and reduced the overall accuracy of CD surveillance. Key quotes are presented in Table 3, with additional supporting quotes presented in Supplementary Table S6.

#### Theme three: data quality control, use, and feedback loops

##### Current data quality control practices

Data quality control practices were largely ad hoc and manual. At the local levels, data entry and reporting relied on manual processes, including the use of paper forms or Excel, and sending results as a WhatsApp photo of the handwritten form or via email. Verification typically occurred at municipal or regional levels, yet errors frequently persisted into national datasets. Informal reporting channels, such as WhatsApp, introduced issues with illegibility and incomplete information, while adherence to mandatory reporting guidelines varied by individual capacity and workload. In Paraguay, for example, it was expected that all cases were reported, although participants shared that adherence was uneven and often dependent on individual health worker initiative and bandwidth to complete the reporting procedure.

##### Data use and feedback across health system levels

Participants described limited feedback loops and restricted access to consolidated data. While epidemiological bulletins were used to promote transparency, data were often presented without interpretation guidance or were incomplete. In Bolivia, participants shared that the MSyD publishes bulletins on either a monthly or quarterly basis, depending on the type of data and urgency to share results. Additionally, participants shared that while there was access to CD data through SUIS, not all data was presented to the public, such as laboratory data. In Paraguay, participants shared that monthly or even weekly epidemiological bulletins were widely used and shared with both regional and local levels. However, some participants expressed that there was a noted lack of visibility into national datasets unless they were formally published or requested, limiting the utility of data for frontline decision-making and local program improvement. Key quotes are presented in Table 3, with additional supporting quotes presented in Supplementary Table S7.

#### Theme four: cross-border data sharing

##### Current practices and barriers

Cross-border data sharing was limited and largely informal, occurring primarily through regional meetings and professional exchanges. In Bolivia, for example, cross-border information was mostly exchanged at the national level, with limited engagement from subnational staff.

Participants described regulatory barriers, mistrust, and limited transparency as key obstacles to systematic data exchange, despite a strong interest in collaboration. Several participants, for example, expressed that they had faced previous challenges when it came to data sharing with other countries, even when it could benefit public health strategies across borders. At the regional level, participants from both countries expressed frustration that, while being responsible for data generation, national level data was not easily accessed.

##### Shared vision for regional data collaboration

Participants expressed a shared vision for an interoperable, standardized regional platform to facilitate cross-country CD data exchange. Priority data included clinical, epidemiological, and entomological information, alongside mechanisms for sharing best practices in case management, diagnostics, and vector control. Furthermore, there was a shared need for standardized indicator lists, which would help countries to align strategies and conduct comparative analyses. Key quotes are presented in Table 3, with additional supporting quotes presented in Supplementary Table S8.

### Cross-cutting synthesis of participant-suggested recommendations for improving data management

Key recommendations were provided across interviews, targeted at health authorities working at the national level in both countries as well as for other global technical staff working in the infectious disease space (Table 4). Participants emphasized the need for a centralized, interoperable HMIS for CD with offline functionality, integration of laboratory and blood bank data, standardized indicators, enhanced frontline training, improved feedback mechanisms, and expanded regional collaboration to strengthen surveillance and data use across the CD data lifecycle. Key quotes are presented in Table 3, with additional supporting quotes presented in Supplementary Table S9.

**Table 4:** Key recommendations for improving CD data management.

Category	Recommendations
For improved system-level CD data management and integration	1) Develop a centralized health management information system (HMIS) for CD (with offline interoperability, if possible) 2) Develop a set of standardized, core indicators for national and departmental reporting 3) Improve data collection practices at the local/facility and municipality levels by reducing variability in tools/platforms used and moving away from paper-based reporting as early as possible 4) Improve linkage of data from blood banks and laboratories with routine CD data through integration with centralized HMIS
For improved data use, feedback loops, and transparency	1) Improve coordination between national programs and departments to confirm roles, identify bottlenecks in data reporting, and data ownership 2) Promote timelier data reporting and more frequent data validation processes between levels 3) Use existing bulletins more effectively and/or explore public-facing dashboards to share data back down to local levels and close feedback loops
For increased capacity building and resourcing	1) Ensure local-level personnel have sufficient resources to properly collect and report on CD data (internet, computers, paper for reporting forms) 2) Provide additional training to expand technical skills of local and municipal level staff (statistical training and training on reporting forms) 3) Provide additional staff to ensure that teams at each level are able to prioritize CD data collection and reporting among other competing responsibilities 4) Prioritize resourcing to clinics/facilities in rural areas which are most endemic for CD
For reaching vulnerable populations and promoting continual case management	1) Prioritize educational outreach and testing/treatment programs to vulnerable groups that are most affected by the disease (i.e., indigenous communities, pregnant women) 2) Improve longitudinal follow-up for CD case monitoring among vulnerable groups
For better cross-program and cross-country data integration	1) Use learnings from other health areas (HIV/TB/COVID-19/Dengue) to improve CD data management, with a general need for increased prioritization of Chagas amongst other disease programs 2) Identify a set of standardized, core indicators (both epidemiological and entomological) that can be shared by Chagas Programs across endemic countries and brainstorm methods for effective sharing (accessible platform, yearly report, etc) 3) Promote cross-departmental and cross-country exchanges to provide trainings, share best practices, and identify challenges in CD data collection and sharing

## Discussion

Throughout interviews, participants stressed the need for a unified, digital HMIS for CD data management. This includes a system that is integrated across levels (local to national) and supports entities that report on routine surveillance data (blood banks and laboratories) with features such as standardized reporting indicators and harmonized data collection and reporting practices. Currently used by the WHO and a few partner organizations, expanding use of DHIS2 or a similar platform to the local levels of CD endemic countries would hold many benefits for national programs like ease of use, standardization, and improving data quality.^21^^,^^22^ Additionally, the expansion of this system would allow for a set of core indicators to be collected and reported on, starting from the core facility levels.^23^ Other endemic countries are considering policy changes to promote data management, such as Brazil, through the implementation of compulsory reporting procedures for chronic CD into the SINAN (Sistema de Informação de Agravos de Notificação, or Information System for Notifiable Diseases), promoting the overall establishment of historical patient data.^24^

Findings also highlight the need for better data communication, accountability, and uptake practices, with a focus on promoting feedback loops and data transparency across levels. While the current use of bulletins works well for some participants, this resource is not routinely available and summarized data is not easily understood by participants from all backgrounds. Furthermore, by not purposefully closing the feedback loop and sharing this information back down to the lower levels, national Chagas Programs are missing out on a key opportunity to improve the quality of their data and motivate their data collectors. In a 2015 study, for example, authors found that the use of an enhanced surveillance program and implementation of a data feedback loop for a malaria program in Zambia led to increased malaria testing rates and a lower number of unconfirmed malaria cases.^25^ In the context of Chagas, this is key to building a more thorough and accurate longitudinal database of routine surveillance data.

Findings stressed the need for increased capacity building and resourcing for CD programs, especially at the local level, with the need for better prioritization for Chagas Programs amongst other competing priorities. Arranging training opportunities, both in and between countries, as well as providing basic resources (reporting forms, an extra computer, etc.) were noted as excellent ways for national programs to fuel momentum across HCWs in local levels and promote skills building. A 2021 study by Emeto and co-workers, for example, found that primary HCWs who had readily available reporting forms in their facilities were three times more likely to report.^26^ Meanwhile, those who received training on disease surveillance reporting and those who received feedback on a previously reported disease were three and four times more likely, respectively, to report their data compared to groups who did not receive such training and supervision opportunities.

The impact of work overload and role strain have also been noted to affect both the mental health of the HCWs as well as hinder disease surveillance and control efforts, such as through poor record keeping, insufficient reporting, and reduced role competence.^27^^,^^28^ With limited resources available for Chagas Programs, it is crucial that the needs of HCWs at the local level are prioritized in resource allocation to prevent these key issues. Additional resourcing to better include marginalized and indigenous groups in both educational outreach and testing/treatment campaigns can also improve the quantity and quality of routine data collected where the disease is most prevalent, as knowledge gaps regarding CD transmission and prevention practices among these populations can limit their health seeking behaviors and touch points with facilities which collect routine data.^29^^,^^30^

Lastly, participants expressed the desire for improved data sharing pathways across endemic countries, with a call to action for other global players in the infectious disease space for improved CD prioritization and resourcing. Developing a set of standardized indicators for CD reporting is a core recommendation, applicable to all endemic countries beyond Bolivia and Paraguay. For example, these findings are also relevant to other cross-border systems in Latin America, such as the Honduras–Guatemala–El Salvador and Venezuela–Colombia–Brazil regions, where coordinated surveillance and data sharing are essential for addressing shared transmission dynamics. While some organizations, such as the Infectious Disease Data Observatory (IDDO) or the Chagas Coalition have aimed to prioritize the visibility of CD data, the available data are often focused on treatment efficacy and diagnostics instead of routinely collected epidemiological or entomological indicators. Additionally, unlike the yearly World Malaria Report (WMR) which presents detailed findings on disease burden, any cross-country data for CD is grouped into a report with other NTDs, with outdated estimates and indicators mainly focused on disease transmission as set by the WHO’s “Compendium of indicators for monitoring and evaluating progress of the road map for neglected tropical diseases 2021–2030”.^31^^,^^32^ Many studies have noted this need for improved global public health surveillance for NTDs, with standardized and transparent data sharing as a key step towards disease prioritization.^33^, ^34^, ^35^

These findings are also relevant to other NTDs beyond Chagas, such as leishmaniasis and dengue. For example, disease burden estimates for leishmaniasis vary widely due to labor intensive reporting procedures, underreporting, and fragmented surveillance systems that limit the availability of reliable routine data.^36^^,^^37^ Similarly, although dengue programs were described by participants as receiving greater funding and surveillance attention in Bolivia and Paraguay compared to Chagas, routine dengue surveillance data across Latin America remain affected by comparable challenges identified in our interviews, including underreporting and failures in compulsory notification.^38^ Collectively, these challenges reinforce the need for integrated cross-border surveillance systems to improve data quality and enable coordinated responses across the Americas.

This study has several limitations. First, there was unequal representation of participants across countries and healthcare system levels. While multiple participants were recruited from the national level in Paraguay, only one participant ultimately agreed to be interviewed from the national level in Bolivia, largely due to the need for employer approval at the time of recruitment. In total, three additional national-level participants (one from Paraguay and two from Bolivia) were invited but did not complete an interview. This limited participation, particularly in Bolivia, may have introduced informational bias and restricted the range of perspectives captured at higher levels of the health system. Although a breadth of topics was captured across levels, the depth of perspectives may not have been fully explored, as sampling was limited to two to three departments in each country. As such, the findings may not be fully representative of the current state of CD data management systems in other endemic settings. Additionally, this study focused primarily on vector-borne chronic CD within routine surveillance systems and may not capture challenges related to other transmission pathways. Finally, as only one member of the research team conducted the interviews, the analysis and interpretation of findings may be limited in scope; however, this was mitigated through the involvement of additional team members with experience in CD research in both countries. While interviews were conducted both in person and virtually, no substantive differences in response depth or thematic content were observed between interview modes.

### Conclusions

While there are many obstacles to overcome to improve CD data management, small steps taken can provide great changes for data transparency, standardization, and CD prioritization. This paper highlights the urgent need for national and international actors to invest in stronger, more integrated CD data management systems to strengthen disease monitoring and evaluation, improve disease surveillance, and promote equitable resource allocation. Future research can explore perspectives of stakeholders in other bordering endemic countries, such as Argentina and Brazil, as well as across additional cross-border systems in Latin America, to inform more effective pathways for data sharing and exchanges. Future research should also expand to include other transmission pathways, such as congenital transmission and acute outbreaks (e.g., oral transmission), which may present distinct data management challenges. Finally, future work should consider questions of CD data ownership and governance to inform appropriate and context-specific stewardship models.

## Contributors

Concept and design: MS, SAR, MRC, JAP; Creation and revision of study tools: MS, SAR, ILG, WC, MRC, JAP; Participant recruitment: MS, WC, MRC, MRS, MM, VL; Data collection: MS; Formal analysis and triangulation: MS, EP, LT; First draft of manuscript: MS; Additional manuscript revisions and final approval: MS, EP, LT, SAR, ILG, WC, MRS, VL, ER, MM, RJM, MRC, JAP; Supervision: JAP, RJM.

## Data sharing statement

Full qualitative transcripts are not publicly available due to the risk of participant identification. De-identified data may be shared on reasonable request for research purposes, subject to ethical approval, data use agreements, and safeguards to protect participant confidentiality.

## AI use statement

During the preparation of this work, the authors used ChatGPT (OpenAI, GPT-5.2) to assist with editing and improving the clarity and readability of the manuscript. After using this tool, the authors reviewed and edited the content as needed and take full responsibility for the content of the published article.

## Editor note

The Lancet Group takes a neutral position with respect to territorial claims in published maps and institutional affiliations.

## Declaration of interests

The authors declare no competing interests.

## References

[1] Pan American Health Organization (2018). https://iris.paho.org/handle/10665.2/68811.

[2] Cucunubá Z.M., Gutiérrez-Romero S.A., Ramírez J.-D. (2024). The epidemiology of Chagas disease in the Americas. Lancet Reg Health Am.

[3] Cousin E., Nascimento B.R., Whisnant J.L. (2025). Global, regional, and national burden of Chagas disease, 1990–2023: a systematic analysis for the Global Burden of Disease Study 2023. Lancet Infect Dis.

[4] Pan American Health Organization (2022). https://www.paho.org/en/news/13-4-2022-less-10-those-infected-chagas-disease-receive-timely-diagnosis-and-treatment‌.

[5] Gómez-Ochoa S.A., Rojas L.Z., Echeverría L.E., Muka T., Franco O.H. (2022). Global, regional, and national trends of Chagas disease from 1990 to 2019: comprehensive analysis of the Global Burden of Disease Study. Glob Heart.

[6] Gürtler R.E. (2009). Sustainability of vector control strategies in the Gran Chaco Region: current challenges and possible approaches. Mem Inst Oswaldo Cruz.

[7] Hopkins T., Gonçalves R., Mamani J., Courtenay O., Bern C. (2019). Chagas disease in the Bolivian Chaco: persistent transmission indicated by childhood seroscreening study. Int J Infect Dis.

[8] Ministerio de Salud y Deportes (BO) (2024). Dirección General de Epidemiología, Unidad de Vigilancia Epidemiológica y Salud Ambiental. Boletín epidemiológico: Semana Epidemiológica No. 27/2024 [Internet].

[9] Gonzalez-Britez N.E., Alevi K.C.C., Caris Garcia A.C., Martínez Purroy C.E., Galvão C., Carrasco H.J. (2021). Chagas disease vectors of Paraguay: entomoepidemiological aspects of Triatoma sordida (Stål, 1859) and development of an identification key for Paraguayan triatomines based on cytogenetics data. Am J Trop Med Hyg.

[10] Ardiles-Ruesjas S., Lesmo V., González-Romero V. (2025). Prevalence and diagnostic accuracy of different diagnostic tests for Chagas disease in an indigenous community of the Paraguayan Chaco. PLoS Negl Trop Dis.

[11] World Health Organization (2025). https://www.who.int/news-room/fact-sheets/detail/chagas-disease-(american-trypanosomiasis).

[12] Kohl S. (2018). OECD-delivering quality health services: a global imperative. Eur J Hosp Pharm Sci Pract.

[13] Koumamba A.P., Ngoungou E.B., Diallo G. (2024). Data quality in a routine health information system: the situation in Gabon as seen from two health regions. Open J Public Health.

[14] World Health Organization- Global Malaria Programme Digital solutions for malaria surveillance. http://www.who.int.

[15] Muniz J., José C. (2024). The mosquito knows no borders: regional challenges for global confrontation in the dengue battle. PLoS Neglected Trop Dis.

[16] Avaria A., Ventura Garcia L., Sanmartino M., Van der Laat C. (2021). Population movements, borders, and chagas disease. Mem Inst Oswaldo Cruz.

[17] World Health Organization (2021). http://www.who.int.

[18] Tong A., Sainsbury P., Craig J. (2007). Consolidated criteria for reporting qualitative research (COREQ): a 32-item checklist for interviews and focus groups. Int J Qual Health Care.

[19] Palinkas L., Horwitz S., Green C., Wisdom J., Duan N., Hoagwood K. (2015). Purposeful sampling for qualitative data collection and analysis in mixed method implementation research. Adm Pol Ment Health.

[20] Moser A., Korstjens I. (2017). Series: practical guidance to qualitative research. part 3: sampling, data collection and analysis. Eur J Gen Pract.

[21] Ndlovu K., Mauco K.L., Keetile M. (2022). Acceptance of the District Health Information System version 2 platform for malaria case-based surveillance by health care workers in Botswana: Web-based survey. JMIR Form Res.

[22] Moungui H.C., Nana-Djeunga H.C., Nko’Ayissi G.B., Sanou A., Kamgno J. (2021). Mixed-methods evaluation of acceptability of the District Health Information Software (DHIS2) for neglected tropical diseases program data in Cameroon. J Glob Health Rep.

[23] Ortu G., Williams O. (2017). Neglected tropical diseases: exploring long term practical approaches to achieve sustainable disease elimination and beyond. Infect Dis Poverty.

[24] da Rocha Siriano L., Marchiol A., Pereira Certo M., Cubides J.C., Forsyth C., Augusto de Sousa F. (2020). Mandatory notification of chronic chagas disease: confronting the epidemiological silence in the State of Goiás, Brazil. Trop Med Infect Dis.

[25] Chisha Z., Larsen D.A., Burns M. (2015). Enhanced surveillance and data feedback loop associated with improved malaria data in Lusaka, Zambia. Malar J.

[26] Emeto D.C., Salawu A.T., Salawu M.M., Fawole O.I. (2021). Recognition and reporting of neglected tropical diseases by primary health care workers in Ibadan, Nigeria. Pan Afr Med J.

[27] Hailu A., Gebre T., Seife F. (2025). Challenges and strategies for mainstreaming neglected tropical diseases campaign interventions in Ethiopia. Am J Trop Med Hyg.

[28] Salam S.P., Ameh C., Dahiru T. Evaluation of the neglected tropical diseases surveillance. https://www.medtextpublications.com/open-access/evaluation-of-the-neglected-tropical-diseases-surveillance-system-in-kaduna-1228.pdf.

[29] Salm A., Gertsch J. (2019). Cultural perception of triatomine bugs and Chagas disease in Bolivia: a cross-sectional field study. Parasit Vectors.

[30] Arrom-Suhurt C.M., Arrom-Suhurt C.H., Arrom-Suhurt M.A., Rolón M., Vega-Gómez M.C., Rojas A. (2018). Socioeconomic profile and perceptions of Chagas disease in indigenous communities of the Paraguayan Chaco. J Public Health.

[31] Global report on neglected tropical diseases 2024. https://www.who.int/teams/control-of-neglected-tropical-diseases/global-report-on-neglected-tropical-diseases-2024.

[32] (2023). A compendium of indicators for monitoring and evaluating progress of the road map for neglected tropical diseases 2021–2030.

[33] Edelstein M., Lee L.M., Herten-Crabb A., Heymann D.L., Harper D.R. (2018). Strengthening global public health surveillance through data and benefit sharing. Emerg Infect Dis.

[34] Tsueng G., Cano M.A.A., Bento J. (2023). Developing a standardized but extendable framework to increase the findability of infectious disease datasets. Sci Data.

[35] Aya Pastrana N., Beran D., Somerville C., Heller O., Correia J.C., Suggs L.S. (2020). The process of building the priority of neglected tropical diseases: a global policy analysis. PLoS Negl Trop Dis.

[36] Romero G.A.S., Boelaert M. (2010). Control of visceral leishmaniasis in Latin America—A systematic review. PLoS Neglected Trop Dis.

[37] Bern C., Maguire J.H., Alvar J. (2008). Complexities of assessing the disease burden attributable to leishmaniasis. PLoS Neglected Trop Dis.

[38] Lessa C.L.S., Fiuza B.S.D., Hodel K.V.S., Minafra C., de Souza Gonçalves M., Machado B.A.S. (2025). Understanding Dengue underreporting: an analysis of the impacts for the world, Latin America and Brazil. Sci World J.

