# Supplementary material for: Strengthening health management information systems for Chagas disease: a multi-level qualitative study in Bolivia and Paraguay
Source: Lancet Reg Health Am. 2026 May 30;60:101514. doi: 10.1016/j.lana.2026.101514 (PMC13241644; doi:10.1016/j.lana.2026.101514)
Supplement: Supplementary Tables S1–S9 [file mmc1.docx]

**Supplementary Material**

**Table of Contents**

[Table S1. Inclusion and exclusion criteria 1](#_heading=h.qseppxjj08t8)

[Table S2. Interview guide (English Copy) 1](#_heading=h.ol7cjxl1mbny)

[Table S3. Participant demographics for key informants in Bolivia 3](#_heading=h.5je4ljqz8g39)

[Table S4. Participant demographics for key informants in Paraguay 4](#_heading=h.vd7k01baluy5)

[Table S5. Additional supporting quotes from key informants for Theme One (Fragmented Chagas Data Systems Across the Data Lifecycle) 5](#_heading=h.5vafr4ksip14)

[Table S6. Additional supporting quotes from key informants for Theme Two (Barriers to High Quality Routine Data) 7](#_heading=h.2613hive0a2f)

[Table S7. Additional supporting quotes from key informants for Theme Three (Data Quality Control, Use, and Feedback Loops) 9](#_heading=h.wsa9ayedcjfa)

[Table S8. Additional supporting quotes from key informants for Theme Four (Cross-Border Data Sharing) 9](#_heading=h.gpg2aupuilzv)

[Table S9. Additional supporting quotes from key informants on recommendations for improving data management 10](#_heading=h.btn1anhmvzuo)

#

# Table S1. Inclusion and exclusion criteria

| **Inclusion** | **Exclusion** |
| --- | --- |
| - Be 18 years or older - Currently work or have worked in the following roles: healthcare personnel (nurse, medical doctor, biochemist), data manager/statistician, technical advisor, epidemiologist, health program director. - Reside in the selected sampling locations in Bolivia or Paraguay. - Currently work or have worked with Chagas data in the past year - Consent to study participation. | - None |

#

# Table S2. Interview guide (English Copy)

| **Participant Name** | [Insert] |
| --- | --- |
| **Date** | [Insert] |
| **Location** | [Insert] |
| **Informed Consent Description**  Hello (name)! My name is (name). I am part of a team of researchers affiliated with the Barcelona Institute for Global Health (ISGlobal) and (insert local partner for country of interview). Thank you for taking time out of your busy schedule to be here with me today.  The purpose of this interview is to learn more about your experience in collecting and reporting data on Chagas disease, as well as to gather your opinions on how to improve Chagas disease data management. The information collected in these interviews will be used to update the Chagas disease reporting pathway and to develop recommendations for a more efficient data management process.  I will take notes and record our conversation as another way of documenting the discussion. All information collected here is strictly confidential and will be destroyed at the end of the study. Participation is voluntary, and you may withdraw at any time during the interview.  Here is a copy of the consent form, which provides a description of the study and the potential benefits/consequences of participating [Provide a copy for participants to read]. Do you have any questions?  Do we have your consent to be interviewed? [Have participant sign the form]  Do we have your consent for the interview to be audio recorded? [Start recording] | |
| **I. General Information**  To start with a fun question: If you could spend a day doing anything you want, what would you do?  Now I will ask some background questions about your role and your previous experience working with Chagas disease.   - What is your profession? - What is your current role? - Can you briefly describe your daily tasks? - How many years have you worked in this position? - Overall, how many years have you worked on projects related to Chagas disease? - Can you briefly describe these projects?   For healthcare personnel:   - How many years have you worked with patients with Chagas disease?   For blood bank/laboratory staff:   - How many years have you worked with positive *T. cruzi* test results? | |
| **II. Experience in Chagas Disease Data Management**  [If they have experience with data collection]   - Can you describe your experience collecting clinical data on Chagas disease? - What type of data do you collect? (e.g., cases) - What challenges, if any, have you encountered when collecting clinical data on Chagas disease?   [If they have experience with data reporting]   - Can you describe your experience reporting clinical data on Chagas disease? Probe: What type of data do you report? (e.g., cases) To whom do you report? How frequently is data reported? - What challenges, if any, have you encountered when reporting clinical data on Chagas disease? - How familiar are you with the current form used to report a positive case? [Have a copy available] - Can you describe the process of reporting a case, starting with completing the form? Probe: What are the main strengths of this form? What are the main limitations of this form? In what ways could the form be improved, if at all?   [If they have additional experience reviewing/storing data]   - Can you describe your experience managing clinical data on Chagas disease (e.g., storage, cleaning, analysis)? - What challenges, if any, have you faced in managing clinical data on Chagas disease? | |
| **III. Integration with Other Health Areas**   - Aside from Chagas disease, do you currently work in any other health areas? (e.g., dengue, leishmaniasis, screening of pregnant women, etc.) - Can you briefly describe how data is collected, reported, and managed in these other health areas? - What lessons, if any, can be drawn from these areas to improve Chagas disease data management? - What measures can be taken to better integrate Chagas disease data management into a general national reporting system? | |
| **IV. Data Sharing**  [Primarily for national-level interviews]   - How is Chagas disease data shared across the country, if at all? - How is Chagas disease data shared with other affected countries, such as neighboring Bolivia, Brazil, or Argentina? - In your opinion, what would be needed to create a cross-country data sharing system? - What type of information is most valuable to share? (e.g., clinical data, entomological data, etc.) | |
| **V. Conclusion**   - Is there anything else you would like to share with us about any of the topics we discussed today?   Thank you for participating in this study. If you have any questions or would like to be informed of the study results, please feel free to email xxx, a member of the team, at xxx. | |

#

# Table S3. Participant demographics for key informants in Bolivia

| **ID** | **Location** | **Organization Type** | **Urban/ Rural** | **Role** | **Years working w/ Chagas data** | **Interview Format** |
| --- | --- | --- | --- | --- | --- | --- |
| **Local/ Facility Level (n=8)** | | | | | | |
| B1 | Yacuiba | Hospital/ Clinic | Rural | Medical doctor | <5 years | In-person |
| B2 | Yacuiba | Hospital/ Clinic | Rural | Medical doctor | <5 years | In-person |
| B3 | Yacuiba | Hospital/ Clinic | Rural | Medical doctor | 10 to <15 years | In-person |
| B4 | Yacuiba | Hospital/ Clinic | Rural | Statistician | <5 years | In-person |
| B5 | Yacuiba | Hospital/ Clinic | Rural | Biochemist | 10 to <15 years | In-person |
| B6 | Yacuiba | Hospital/ Clinic | Rural | Biochemist | 15+ years | In-person |
| B7 | Yacuiba | Hospital/ Clinic | Rural | Medical doctor | 10 to <15 years | In-person |
| B8 | Tarija | Hospital/ Clinic | Urban | Biochemist | <5 years | In-person |
| **Municipality/ District Level (n=5)** | | | | | | |
| B9 | Yacuiba | Government | Rural | Public health staff/ data manager | 5 to <10 years | In-person |
| B10 | Yacuiba | Red de Salud | Rural | Statistician | 10 to <15 years | In-person |
| B11 | Yacuiba | Red de Salud | Rural | Epidemiologist | 10 to <15 years | In-person |
| B12 | Santa Cruz | Red de Salud | Urban | Statistician | 1 to <5 years | In-person |
| B13 | Santa Cruz | Red de Salud | Urban | Nurse/ data manager | <5 years | In-person |
| **Departmental Level (n=11)** | | | | | | |
| B14 | Tarija | SEDES | Urban | Technical advisor | 15+ years | In-person |
| B15 | Tarija | SEDES | Urban | Biochemist | 15+ years | In-person |
| B16 | Tarija | SEDES/ SUIS* | Urban | Data manager | 15+ years | In-person |
| B17 | Tarija | SEDES | Urban | Medical doctor/ Technical advisor | <5 years | In-person |
| B18 | Tarija | SEDES | Urban | Medical doctor/ Program staff | 15+ years | Online |
| B19 | Tarija | Blood Bank | Urban | Medical doctor | 5 to <10 years | In-person |
| B20 | Santa Cruz | Programa de Chagas | Urban | Statistician | 5 to <10 years | In-person |
| B21 | Santa Cruz | Programa de Chagas | Urban | Technical advisor | 15+ years | In-person |
| B22 | Santa Cruz | SUIS* | Urban | Statistician | 15+ years | In-person |
| B23 | Sucre | Programa de Chagas | Urban | Medical doctor/ Program staff | 15+ years | Online |
| B24 | Sucre | Programa de Chagas | Urban | Medical doctor | 15+ years | Online |
| **National Level (n=1)** | | | | | | |
| B25 | La Paz | Ministry of Health/ SUIS* | Urban | Medical doctor/ Technical advisor | 15+ years | Online |

**SUIS was formally referred to as the “SNIS” or “Sistema Nacional de Informacion de Salud”*

# Table S4. Participant demographics for key informants in Paraguay

| **ID** | **Location** | **Organization Type** | **Urban/ Rural** | **Role** | **Years working w/ Chagas data** | **Interview Format** |
| --- | --- | --- | --- | --- | --- | --- |
| **Local/ Facility Level (n=8)** | | | | | | |
| P1 | Asunción | Hospital/ Clinic | Urban | Medical doctor | 15+ years | In-person |
| P2* | Asunción | Hospital/ Clinic | Urban | Medical doctor | <5 years | Online |
| P3 | Irala Fernández | Hospital/ Clinic | Rural | Nurse | <5 years | In-person |
| P4 | Irala Fernández | Hospital/ Clinic | Rural | Medical doctor | <5 years | In-person |
| P5 | Irala Fernández | Hospital/ Clinic | Rural | Medical doctor | <5 years | In-person |
| P6 | Campo Aceval | Hospital/ Clinic | Rural | Nurse | <5 years | In-person |
| P7 | El Estribo | Hospital/ Clinic | Rural | Medical doctor | <5 years | In-person |
| P8 | Casanillo | Hospital/ Clinic | Rural | Nurse | <5 years | In-person |
| **Municipality/ District Level (n=1)** | | | | | | |
| P9 | Irala Fernández | Hospital/ Clinic | Rural | Nurse/ Data manager | 10 to <15 years | In-person |
| **Departmental/ Regional Level (n=4)** | | | | | | |
| P10 | Capital District (Asunción) | Blood Bank | Urban | Medical doctor | 15+ years | In-person |
| P11 | Capital District (Asunción) | Blood Bank | Urban | Medical doctor | 15+ years | In-person |
| P12 | Presidente Hayes | Ministerio de Salud Pública y Bienestar Social-XV Región Sanitaria | Urban | Epidemiologist/ Data manager | 5 to <10  years | Online |
| P13 | Irala Fernández | Laboratorio Central de Salud (Central Reference Laboratory) | Rural | Biochemist | <5 years | In-person |
| **National Level (n=5)** | | | | | | |
| P14* | Capital District (Asunción) | Laboratorio Central de Salud (Central Reference Laboratory) | Urban | Biochemist | 15+ years | In-person |
| P15* | Asunción | Ministry of Health | Urban | Program staff | 15+ years | In-person |
| P16 | Asunción | SENEPA | Urban | Medical doctor/ Program staff | 15+ years | In-person |
| P17 | Asunción | SENEPA | Urban | Medical doctor/ Program staff | 15+ years | In-person |
| P18 | Asunción | SENEPA | Urban | Statistician | <5 years | In-person |

#

**Participants marked with an asterisk correspond to the initial pilot interviews (n=3), after which the interview guide was revised; these interviews were included in the final analysis*.

#

#

# Table S5. Additional supporting quotes from key informants for Theme One (Fragmented Chagas Data Systems Across the Data Lifecycle)

| **Subtheme** | **#** | **Quote** | **Participant** |
| --- | --- | --- | --- |
| Data collection across data sources and levels | 1 | “We prepare data based on work in different municipalities. We have 10 municipalities with vinchuca presence and control. The main data handled are entomological evaluations of intra- and peri-domicile infestations to see the presence or absence of vinchucas. Each municipality has an annual program…how many houses to evaluate or spray per year…and a table shows compliance. We monitor infestation levels month by month.” | Municipality Level, Bolivia |
|  | 2 | “...Cases, pediatric clinical cases…Age, origin, sex, history of the vinchuca vector sightings and physical examination, and so on.” | Facility Level, Paraguay |
| Data flow pathways and reporting systems (Bolivia) | 3 | “Every 5th of every month all the information is collected and consolidated first. There is a form. The information is extracted in a type of file and consolidated in a system called SUIS. And the laboratory information is consolidated… everything is kind of included there, it is sent as a package to the health network. It is also sent to them in printed form.” | Facility Level, Bolivia |
|  | 4 | “[The data] arrives until the 10th of each month here. After that, as a department, there is a deadline of the 18th of each month, when it is then sent to the national level. The national level uploads it from there on a web platform.” | Departmental Level, Bolivia |
|  | 5 | “We handle forms provided by the national [Chagas Program]. The SUIS is the health information system where we’re supposed to have all this data, but I’d like it to contain the same information we handle in the forms. Every month, we must compare SUIS data with our forms, which takes time. Sometimes centers enter information in SUIS but don’t send it to us or send it to us but don’t enter it in SUIS.” | Departmental Level, Bolivia |
|  | 6 | “This year the health information system has undergone a modification, and new variables have been entered into the system. We are currently having conflicts with the issue of transfers, because our municipality no longer uses manual notebooks…we have entered a process of digitization of the production notebooks. So, all the records are made digitally in the primary health care software, except for some programs that continue to use the physical medium.” | Facility Level, Bolivia |
|  | 7 | “Each program receives information from the health networks…, what it does is to compile all that information and in turn it is also uploaded through the epidemiological files to the SUIS and the SUIS proceeds to the report, whether it is immediate, weekly or monthly, to the National Information System. So, there is a parallel information that is both in the program for management and control, taking preventive actions above all, and there is also the part that goes to the SUIS, which is to upload it to the National Information System.” | Departmental Level, Bolivia |
|  | 8 | “Within the health information system, information is generated in health facilities…hospitals in Yacuiba or elsewhere…based on clinical histories and epidemiological records. This data is systematized in hospital notebooks, consolidated in Form 302A, and sent to health networks. The networks perform quality control and send the consolidated data to SEDES. SEDES reviews and sends departmental data to the Ministry of Health, which performs final quality control and publishes it on the Ministry’s website.” | National Level, Bolivia |
| Data flow pathways and reporting systems (Paraguay) | 9 | “In my department, as my [health facility] is far from the health region, what we do is to complete the notification form, scan it and send it for surveillance, which would be in the health region. Then the notification goes in physical form, once I submit my reports, and it goes with the reports.” | Facility Level, Paraguay |
|  | 10 | “We collect the data here and there is a main person in charge at the health center… there is a template form provided by the Ministry and it is used everywhere.” | Facility Level, Paraguay |
|  | 11 | “We work with SENEPA. When SENEPA identifies a notification that has not been entered into the system, they share that notification with us so that it can be officially entered. So this is a bit of our daily work with Chagas disease notification, training health personnel to make them aware that [Chagas], like other diseases, has mandatory notifications that have to follow the same flow.  Up until now, notification for all the [disease programs] that are part of the National Surveillance System is done on a paper form that is completed. This paper form is scanned- it can be sent by e-mail or any other digital means. It arrives here, at the General Directorate of Health Surveillance, and what we do is to load it into a computer system that we are designing for that purpose.  We are planning to enable users to enter this system for all levels of care by the end of this year. Then, when a suspicion or notification of a case of Chagas disease is detected, the personnel will be able to enter the system from their department and load it directly and not have to fill out a paper form.” | National Level, Paraguay |
| Role of blood banks and laboratories in data collection and reporting | 12 | “What we need is to improve the notification of what blood banks report. We have signed agreements with them, we have validated instruments between the three institutions (SENEPA, the MOH, and blood banks), but it is still difficult to have this information in real time.” | National Level, Paraguay |
|  | 13 | “When we admit [blood] donors, before they come in to donate, they undergo some tests. Among them is the rapid test for Chagas disease. With this rapid test, we can see if the donor is reactive or not [for Chagas]. And if it is reactive, we refer the donor to a place called the Chagas Platform… There is another filter, which is the laboratory. The laboratory performs, within these tests, the Chagas test, in addition to HIV, Hepatitis B, Hepatitis C, Syphilis. So, it is another place where we can collect data from people or donors who are positive for this disease. But the laboratory not only performs the tests in the blood bank- if they are positive, they send [the data] to their department to reconfirm and then inform the donor about the result.” | Departmental Level, Bolivia |
| Consequences of fragmented and parallel reporting systems | 14 | “There is duplication of data, one could say, because, for example, a patient comes to a hospital, they test her, she comes out positive and this hospital reports [the case]. Two months go by and the same patient returns to the hospital. They ask her again for the Chagas test, she comes out positive again, and they continue reporting the same patient twice in the same hospital.  And well, it also happens that this same patient goes to another health center…that health center reports the same patient's data again, so it is as if three patients were infected with Chagas, but the reality is that it is only one patient. So there we have duplicity, even triplicity of information on the same patient. So what we are doing now is to create a database where we can [clean the data] so that the data are more real.” | Departmental Level, Bolivia |
|  | 15 | “The [forms] do not always fit together, and everything is not always compatible. So, if the instruments do not match, they must reprocess the data for us, and that sometimes delays the sending. The fact that we send on the 18th, for example, to the ministry, may mean that some services have been delayed and will arrive later.” | Departmental Level, Bolivia |
|  | 16 | “The health center makes its report and uploads its data to the SUIS program, with the SUIS forms, but it also has to send us its forms based on our formats, because we manage our own formats as a program.” | Departmental Level, Bolivia |

# Table S6. Additional supporting quotes from key informants for Theme Two (Barriers to High Quality Routine Data)

| **Subtheme** | **#** | **Quote** | **Participant** |
| --- | --- | --- | --- |
| Human resource and training constraints | 1 | “The difficulty we have is the quality control of data within the facility, because the facility does not have in their administration, for the most part, people who oversee doing this information transfer and review process.  I will give you an example. There are a doctor and a nurse-they take care of patients, they have to enter information, and they have to review information and send…sometimes, due to time constraints, they do not have enough time to verify and confirm the information. Due to the lack of human and administrative resources, they can make mistakes and have errors.  But the information is never closed- it is always open to correction throughout the year before the year ends. We even have two months after the end of the year to make corrections.” | Municipality Level, Bolivia |
|  | 2 | “I do not have a doctor, so I am in charge of my family health unit. I am the one who does the consultations, everything related to the program-sexual and reproductive health, the national child nutrition program, consultations, etc.” | Facility Level, Paraguay |
|  | 3 | “Many times, the individual is tired when uploading the data. They don't upload all the data, the diagnosis is ready, it's over. And sometimes that day he does not upload it because he was tired or for some other reason, he uploads it after a week…Obviously, the quality was practically lost.” | Facility Level, Bolivia |
|  | 4 | “There has been a lack of knowledge of the health personnel. As there is personnel turnover and new personnel coming in, the implementation of the collection instruments is lost in this process…That has been the most complicated thing. You implement with one staff, a new one comes in, and you have to do it all over again. And that causes a delay in the delivery of information.” | Municipality Level, Bolivia |
| Infrastructure, geography, and access constraints | 5 | “The difficulty that I usually have is, for example, to confirm the positive result that I get within my [health unit]. That is the biggest difficulty I have as my health center is 145 km away from my health unit. So that is what makes it most difficult for us to confirm a positive result.” | Facility Level, Paraguay |
|  | 6 | “[The patient] does not want to take the sample because they see that the blood is being taken, and they think that if they take that little bit of blood it is already a lot of blood. Other participants, for example, have told me that they are afraid because they think we are going to sell the blood too.” | Regional Level, Paraguay |
|  | 7 | “Because of the lack of internet… their equipment where they enter the data has sometimes been fried and there is no one to do maintenance, and so on.... That's why, in the past, it was difficult to get [data] from the rural areas, from the farthest away.” | Departmental Level, Bolivia |
| Implications for data completeness and timeliness | 8 | “After a while, I went back to the municipalities and they no longer had the files saved and I also went back to the NGO where we had left the data and the computers where the information was left had been manipulated, the information had been erased. So, it was a problem to collect all that information, because we tried to follow up on those children that we treated and we could no longer do so because the information was missing. So, that was an initial experience. Later, at the Chagas center, where I worked for a long time, we stored the information in software, specifically designed to be able to have good quality information.” | Departmental Level, Bolivia |
|  | 9 | “There have been cases where a patient has been treated in one department and that same patient has been treated in another department and [the data] does not show that they were treated again. That is why I think it is important, even at a national level, that it should be systematized.” | Departmental Level, Bolivia |
|  | 10 | “Sometimes they give you the basics…the patient's name…sometimes they don't give you the address, they don't give you the phone number…so sometimes it's hard to find that patient…. But even so, the completeness [on the reporting form] is generally not exactly 100%.” | National Level, Paraguay |
| Ad hoc data quality control practices | 11 | “Sometimes they change the names- the technicians may not give me the name how it is or how it is normally spelled. That is a difficulty, let's say, for me, because I almost have to guess what it says, right? But this [information], for example, was sent by WhatsApp, but in order to have this information as soon as possible the person in charge printed it directly and passed it to me.” | Departmental Level, Bolivia |
|  | 12 | “The first levels detect the cases; they report it here to the network. From the network, we report it to the SEDES [Department] and from the SEDES it goes to the Ministry. That would be the way. The difficulty is that sometimes a facility handles the data manually, let's say, or by program, and does not check it against the SUIS, which is the National Information System that integrates all the information from a facility. So, there tends to be no correlation of data.” | Municipality Level, Bolivia |
|  | 13 | “Sometimes what they send by WhatsApp doesn’t match what they send physically. We validate and review the information with the SUIS to see if data from health centers or municipalities coincide. We compare SUIS data with what we receive; if there’s variation—one more house, two fewer, positive or evaluated—we do a timely review. Technicians are called; if it can be solved by phone, it’s done. If not, we check the journal form to find the fault.” | Departmental Level, Bolivia |
|  | 14 | “We have meetings for quality control of the data with the technical chief here at the Chagas Program… and those responsible for surveillance. We are always meeting in that sense…We also emphasize this to the networks, to those responsible for information, that they should watch over and coordinate with the personnel of the different programs.” | Departmental Level, Bolivia |

# Table S7. Additional supporting quotes from key informants for Theme Three (Data Quality Control, Use, and Feedback Loops)

| **Subtheme** | **#** | **Quote** | **Participant** |
| --- | --- | --- | --- |
| Current data quality control practices | 1 | “Sometimes they change the names- the technicians may not give me the name how it is or how it is normally spelled. That is a difficulty, let's say, for me, because I almost must guess what it says, right? But this [information], for example, was sent by WhatsApp, but to have this information as soon as possible the person in charge printed it directly and passed it to me.” | Departmental Level, Bolivia |
|  | 2 | “Sometimes what they send by WhatsApp doesn’t match what they send physically. We validate and review the information with the SUIS to see if data from health centers or municipalities coincide. We compare SUIS data with what we receive; if there’s variation—one more house, two fewer, positive or evaluated—we do a timely review. Technicians are called; if it can be solved by phone, it’s done. If not, we check the journal form to find the fault.” | Departmental Level, Bolivia |
|  | 3 | “We have meetings for quality control of the data with the technical chief here at the Chagas Program… and those responsible for surveillance. We are always meeting in that sense…We also emphasize this to the networks, to those responsible for information, that they should watch over and coordinate with the personnel of the different programs.” | Departmental Level, Bolivia |
| Data use and feedback across health system levels | 4 | “On the Ministry's web page, there are informative bulletins that report on the most important epidemiological data. Sometimes they do it monthly, sometimes on an annual basis. This will depend on the seriousness of the problem.” | Facility Level, Bolivia |
|  | 5 | “The bulletins are sent periodically [on the website] and there you can see the information on all the programs, including Chagas…The number of patients diagnosed and treated and some of the program's own preventive activities.” | Departmental Level, Bolivia |

# Table S8. Additional supporting quotes from key informants for Theme Four (Cross-Border Data Sharing)

| **Subtheme** | **#** | **Quote** | **Participant** |
| --- | --- | --- | --- |
| Current practices and barriers | 1 | “We have meetings at the Mercosur level, at Ibero-American level. And with Argentina we do have a more frequent flow, because blood plasma is industrialized in Argentina. So, there is an epidemiological flow of mandatory data every six months, where we report the tests to these units.” | Regional Level, Paraguay |
|  | 2 | “To the best of my knowledge, yes, we are also sharing data with Bolivia, because we had also done a 15-day training program in Bolivia on the management of Chagas disease…Both on prevention and treatment.” | Facility Level, Paraguay |
|  | 3 | “The data is usually shared in INCOSUR... there is a meeting of all the members of the South every two years. So far we do not have a base like that in which data is uploaded…This is being formed now by PAHO to have a constant base of information. “ | National Level, Paraguay |
|  | 4 | “The fact is that since we handle a health format at the Bolivian level, then at the national level the same guideline should be established to establish an international standard so that we all handle the same thing. So that if I travel to another country my record is similar to that country's record.” | Departmental Level, Bolivia |
| Shared vision for regional data collaboration | 5 | “It is good to have both epidemiological information to know how the levels of infestation, the level of vector control, indicators for congenital transmission…It is also necessary to have clinical data, for example, information on patients themselves. In general, I believe that both are what we need to strengthen [the data system] ...we still do not have all this information consolidated.” | Departmental Level, Bolivia |
|  | 6 | “[We should] make a base of the main indicators that we would need, especially in the region to be able to have those bases and make comparisons… distribution of cases, prevalence, indices, cases among pregnant women… I think these are the strong indicators that we should have for Chagas.” | Regional Level, Paraguay |
|  | 7 | “I visited Paraguay, and I had the opportunity to attend a training there…They asked us to share with them how we can organize the data. In relation to Argentina, Colombia, other places…they manage their information, but they have certain misgivings, they do not share easily. So, it is difficult to share. That is why perhaps it would be good to have a virtual platform where one can have access to statistical data, to places where one can find percentages, prevalence rates, infestation rates in general, etc.… perhaps it is necessary to have this type of information sharing between one country and another. | Departmental Level, Bolivia |
|  | 8 | “We also share certain diseases among countries, especially neighboring countries… the type of T. cruzi in each country is different, so it would be good to know, for example, what is circulating among the neighbors. In other words, these are much finer details than knowing the numbers ... Also, apart from just purely data, always the innovations that each country makes and that have positive experiences can be beneficial [to share] between countries.” | National Level, Paraguay |

# Table S9. Additional supporting quotes from key informants on recommendations for improving data management

| **Subtheme** | **#** | **Quote** | **Participant** |
| --- | --- | --- | --- |
| For improved system-level CD data management and integration | 1 | “For the Chagas program, I think it enters the same system, with its own notification system like TB or HIV. Chagas has its own program, actions, and control. What could be improved is merging with other vector programs to better use technical personnel—since some work only on Chagas or only on dengue, and we often lack technicians. They should be multifunctional to optimize the work.” | Departmental Level, Bolivia |
|  | 2 | “I would like to have an exclusive platform with all the data we need to have robust data, that the information systems and the data loading, everything is online and that we can work with the whole country, that we have access to all the country's media and be able to have an application base that facilitates the analysis of the data.” | National Level, Paraguay |
|  | 3 | “If they had access in real time to the information, when a donor, as he said, or a trainee with an intestinal or cardiac history, if they had the information that we generate, would that be more dynamic or vice versa. In our case also, if there is a previous diagnosis, as in Chagas a chronic disease, a person can be a carrier of apparently healthy, asymptomatic Chagas, for us it will also be useful, especially the cost reduction at the time of identifying these people.” | Regional Level, Paraguay |
|  | 4 | “For Leishmaniasis, they have SIS-Leish, a system where all data are uploaded and managed nationally and internationally with PAHO. The platform allows communication, visualization, correction, and analysis of cases with permanent control. Dengue also has its own system in the General Directorate of Health Surveillance for positive, negative, confirmed, or discarded cases, with constant laboratory and clinical confirmation. Malaria, though eradicated, also maintains its own permanent system. That is what we want for Chagas. Currently, communication is through cards—positive, negative, medicine—but not a well-formed system like the others.” | National Level, Paraguay |
|  | 5 | “Above all, there’s a need to work in an articulated manner between the Chagas program and hospitals. If they had real-time access to information…about donors or patients with intestinal or cardiac history. It would be more dynamic. For us, knowing previous diagnoses of asymptomatic carriers would help reduce costs when identifying cases. Paraguay is already certified free of vector transmission and has a standardized system to avoid transfusion transmission, but not vertical transmission. As our population is young and healthy, eliminating vertical transmission should be a priority, especially among healthy donors.” | Regional Level, Paraguay |
|  | 6 | “We have to improve our relationship with the blood bank, which is an important sector in which there is a lot of information on positive cases to which we do not have access… we need to look for a way in which we can link their positive or negative cases with ours, because it is also a test that is done on a person and we do not know who, how or when.” | National Level, Paraguay |
| For improved data use, feedback loops, and transparency | 7 | “If we have that energy to get that information, why don't we have it in publications… have the data more public and available. You can only get data if you are on a familiar level with someone (aka through a phone call). We are looking for a system where they can visualize data, download data, and have the data available for use.” | Regional Level, Paraguay |
|  | 8 | “Create a communication page, which is a public page where the public has access… I work around the region here, the Gran Chaco. I would like a map I can see in which there is more presence of disease or where we have more control efforts.” | Facility Level, Bolivia |
| For increased capacity building and resourcing | 9 | “If we talk about data collection or data storage, we need to have the equipment in good condition. Also to have an internet signal. And to have the personnel to do that.” | Departmental Level, Bolivia |
|  | 10 | “We emphasize that Chagas, by law, in Paraguay is obligatory notification. Like any other disease, [the health worker/ data manager] would have to fill out the form and they would have to notify us. That is why we have improved-we notify much more, but there is still a long way to go. We are insisting on this in all the training- we take the form to them, and we insist that they must notify us.” | National Level, Paraguay |
|  | 11 | “Here in the blood bank there are different training programs- training between different countries to learn different things. For example, people who want to come here to learn. The people in charge of the Chagas program come from time to time to train the staff to explain to us how [the data] is being managed and the data available.” | Departmental Level, Bolivia |
| For reaching vulnerable populations and promoting continual case management | 12 | “Chagas disease is a concern at least with my population… I have a lot of cases of Chagas with my population…I do not have the means, for example, to get the villagers to go to a laboratory, at least to go to [the health center], so that they can have their laboratory testing done.” | Facility Level, Paraguay |
| For better cross-program and cross-country data integration | 13 | “Bolivia is here, Paraguay is here and Argentina is here…A coordinated work can be done between the departmental health services, perhaps of the regions involved, to combat, for example, to do a spraying work to combat the vinchucas.” | Facility Level, Bolivia |
|  | 14 | “The simplest thing would be to create a WhatsApp group... For example, there are three countries: Argentina, Bolivia and another country that can be in the same WhatsApp group. But it would be a good initiative to create groups, even if it is with the representatives of each program in each country, and to manage more updated information on this disease.” | Facility Level, Bolivia |
